# Effectiveness of a Tai-Chi Training and Detraining on Functional Capacity, Symptomatology and Psychological Outcomes in Women with Fibromyalgia

**DOI:** 10.1155/2012/614196

**Published:** 2012-05-09

**Authors:** Alejandro Romero-Zurita, Ana Carbonell-Baeza, Virginia A. Aparicio, Jonatan R. Ruiz, Pablo Tercedor, Manuel Delgado-Fernández

**Affiliations:** ^1^Department of Physical Education and Sports, School of Sports Sciences, University of Granada, 18011 Granada, Spain; ^2^Department of Physical Education, School of Education Sciences, University of Cádiz, 11519 Puerto Real, Cádiz, Spain; ^3^Department of Physiology, School of Pharmacy, University of Granada, 18071 Granada, Spain; ^4^Unit for Preventive Nutrition, Department of Biosciences and Nutrition, Karolinska Institutet, 14183 Huddinge, Sweden

## Abstract

*Background*. The purpose was to analyze the effects of Tai-Chi training in women with fibromyalgia (FM). *Methods*. Thirty-two women with FM (mean age, 51.4 ± 6.8 years) attended to Tai-Chi intervention 3 sessions weekly for 28 weeks. The outcome measures were: tenderness, body composition, functional capacity and psychological outcomes (Fibromyalgia impact questionnaire (FIQ), Short Form Health Survey 36 (SF-36)). *Results*. Patients showed improvements on pain threshold, total number of tender points and algometer score (all *P* < 0.001). The intervention was effective on 6-min walk (*P* = 0.006), back scratch (*P* = 0.002), handgrip strength (*P* = 0.006), chair stand, chair sit & reach, 8 feet up & go and blind flamingo tests (all *P* < 0.001). Tai-Chi group improved the FIQ total score (*P* < 0.001) and six subscales: stiffness (*P* = 0.005), pain, fatigue, morning tiredness, anxiety, and depression (all *P* < 0.001). The intervention was also effective in six SF-36 subscales: bodily pain (*P* = 0.003), vitality (*P* = 0.018), physical functioning, physical role, general health, and mental health (all *P* < 0.001). *Conclusions*. A 28-week Tai-Chi intervention showed improvements on pain, functional capacity, symptomatology and psychological outcomes in female FM patients.

## 1. Background

Fibromyalgia (FM) is a chronic diffuse pain condition that probably results from abnormal central pain processing [1, 2]. The symptoms most frequently are chronic pain, characterized by generalized pain, stiffness, fatigue, disturbed sleep, psychological distress, and impaired cognitive function [3, 4].

Physical exercise therapy might be an alternative approach [5]. It has been suggested that a nonextenuating physical exercise, mind-body exercise, and some type of relaxation therapies can also increase pain tolerance, producing a global improvement in the quality of life of FM patients [6–9].

Tai-Chi is a balance-based exercise that integrates low-speed and low-impact body movements with deep breathing together with elements of relaxation and mental concentration. It has been shown to improve physical function, strength, and balance, reduce falls in older adults [10], and improves quality of life and psychological health [11–13]. A recent review suggested potential benefits from Tai-Chi exercise on balance and psychological health [14]. In addition, the benefits of Tai-Chi therapy include improvements in physical and mental well-being in patients with a variety of diseases and disorders [15]. As a traditional Chinese style mind-body exercise [16], Tai-Chi practice requires tranquility of mind during slow movements [17, 18]. Tai-Chi is regarded as a light exercise [19, 20] that consists in a series rhythmic movements that emphasize trunk rotation, weight shifting, and coordination [21]. During Tai-Chi practice, diaphragmatic breathing is coordinated with graceful motions to achieve mind tranquility [20].

Three studies analyzed the benefits of Tai-Chi in women and men with FM [22–24], yet more studies are needed to confirm their results. Furthermore, to our knowledge, there are no studies investigating the effect of Tai-Chi long-term intervention in FM patients. Thus, the purpose of the present study was to analyze the effects of a 7-month Tai-Chi training and detraining (3 months) on functional capacity, quality of life, symptomatology, and psychological outcomes in women with FM.

## 2. Materials and Methods

### 2.1. Study Participants and Design

We contacted with two local associations of patients with FM (Granada and Motril, Spain). Thirty-eight potentially eligible patients responded and gave their written informed consent after receiving detailed information about the aims and study procedures. The inclusion criteria were: (i) meeting the American College of Rheumatology (ACR) criteria widespread pain for more than 3 months and pain with 4 kg/cm^2^ of pressure for 11 or more of 18 tender points [4], (ii) not to have other severe somatic or psychiatric disorders, such as stroke or schizophrenia, or other diseases that prevent physical loading, and (iii) no to be attending another type of physical therapy at the same time. After the baseline measurements, 6 patients refused to participate due to incompatibility with job schedule. Therefore, a final sample of 32 women with FM participated in the study. Patients were not engaged in regular physical activity >20 minutes on >3 days/week.

**Figure 1:**
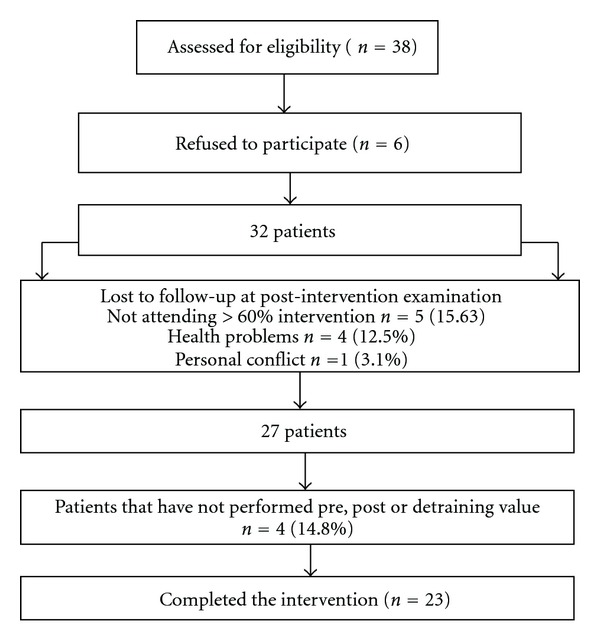
Flow of subjects.

The study flow of patients is presented in Figure 1.

Originally, the aim was to assess a control group of age- and gender-matched patients, but we had an ethical obligation with the Association of Fibromyalgia Patients (Granada, Spain) to provide treatment to all patients willing to participate in the study. Then, a quasi-experimental reversal design was applied, that is, lacking a control group. The purpose of the research design was to determine a baseline measurement, evaluate a treatment (Tai-Chi intervention), and evaluate a return to a nontreatment condition (detraining) in the same group of participants. This type of design particularly controls participant bias well, as the same individual is used at each testing time point. The study outcomes were measured before the intervention (baseline), after 28 weeks of intervention (post-test), and after 3 months of a detraining period (detraining) during which the patients stopped practicing Tai-Chi and did not engage in any structured exercise intervention.

The research protocol was reviewed and approved by the Ethics Committee of the *Virgen de las Nieves Hospital* (Granada, Spain). The study was carried out between November 2009 and September 2010, following the ethical guidelines of the Declaration of Helsinki, last modified in 2000.

### 2.2. Intervention

Patients participated in three 60-minute Tai-Chi sessions conducted weekly for 28 weeks. Each session included: 15 minutes of warmup with stretching, mobility, and breathing techniques; 30 minutes of Tai-Chi exercises principles and techniques, and finally, 15 minutes of various relaxation methods. The intervention consisted of 8 forms from Yang Style Tai- Chi, with minor modifications that were suitable for patients with FM. For example, the first month some exercises were realized with the patients sitting to avoid too much fatigue.

Classes were taught by a Tai-Chi master with teaching experience. The first two weeks of the intervention were focused on learning fundamental movement patterns. In subsequent sessions, patients practiced 8-Form, Yang Style Tai-Chi under master supervision.

Training intensity was controlled by the rate of perceived exertion (RPE) based on Borg's conventional (6–20 point) scale. The medium value of RPE was 11 ± 1. This RPE value corresponds to a subjective perceived exertion of “light,” that is, low intensity.

### 2.3. Outcomes Measures

Pretest, posttest, and detraining intervention assessments were carried out on two separate days with at least 48 hours between each session. This was done in order to prevent patient's fatigue and flare-ups (acute exacerbation of symptoms). The assessment of the tender points, blind flamingo test, chair stand test, and psychological outcomes were completed on the first visit. Body composition and the chair sit and reach, back scratch, 8 feet up and go, handgrip strength, and 6-min walk tests were performed on the second day.

#### 2.3.1. Tender Points Assessment

We assessed 18 tender points according to the American College of Rheumatology criteria for classification of FM using a standard pressure algometer (EFFEGI, FPK 20, Alfonsine, Italy) [4]. The mean of two successive measurements at each tender point was used for the analysis. Tender point scored as positive when the patient noted pain at pressure of 4 kg/cm^2^ or less. The total count of such positive tender points was recorded for each participant. An algometer score was calculated as the sum of the minimum pain-pressure values obtained for each tender point.

#### 2.3.2. Body Composition and Anthropometric Assessment

We performed a bioelectrical impedance analysis with an eight-polar tactile-electrode impedanciometer (InBody 720, Biospace). The validity of this instrument was reported elsewhere [25, 26]. Height (cm) was assessed using a stadiometer (Seca 22, Hamburg, Germany), body mass index (BMI) was calculated as weight (in kilograms) divided by height (in meters) squared. Waist circumference (cm) was measured with the participant standing at the middle point between the ribs and ileac crest (Harpenden anthropometric tape Holtain Ltd).

#### 2.3.3. Functional Capacity

To assess functional capacity we used the Senior Functional Fitness Test Battery [27]. Additionally, we also measured the handgrip strength and the blind flamingo test, which have been used in FM patients [28]. The fitness test battery was administered by trained and qualified researchers.

Lower-body muscular strength: the “Chair stand test” involves counting the number of times within 30 second that an individual can rise to a full stand from a seated position with back straight and feet flat on the floor, without pushing off with the arms [27]. The patients carried out 1 trial after familiarization.

Upper-body muscular strength: “Handgrip strength” was assessed using a digital dynamometer (TKK 5101 Grip-D; Takey, Tokyo, Japan) as described elsewhere [29]. Patients performed (alternately with both hands) the test twice allowing a 1-minute rest period between measures. The best value of 2 trials for each hand was chosen and the average of both hands was registered.

Lower-body flexibility: in the “chair sit and reach test,” the patient seated with one leg extended slowly bends forward sliding the hands down the extended leg in an attempt to touch (or past) the toes. The number of centimeters short of reaching the toe (minus score) or reaching beyond it (plus score) is recorded [27]. Two trials with each leg were measured and the best value of each leg was registered, being the average of both legs used in the analysis.

Upper-body flexibility: the “back scratch test,” a measure of overall shoulder range of motion, involves measuring the distance between (or overlap of) the middle fingers behind the back [27]. This test was carried out alternately with both hands twice, and the best value was registered. The average of both hands was used in the analysis.

Static balance: it was assessed with the blind flamingo test [30]. The number of trials needed to complete 30 seconds of the static position is recorded, and the chronometer is stopped whenever the patient does not comply with the protocol conditions. One trial was accomplished for each leg, and the average of both values was selected for the analysis.

Motor agility/dynamic balance. The “8 feet up and go test” involves standing up from a chair, walking 8 feet to and around a cone, and returning to the chair in the shortest possible time [27]. The best time of two trials was recorded and used in the analysis.

Aerobic endurance: we assessed the “6-min walk test.” This test involves determining the maximum distance (meters) that can be walked in 6 min along a 45.7 meters rectangular course [27, 31].

#### 2.3.4. Psychological Outcomes

Symptomatology was assessed by means of the Spanish version of the Fibromyalgia Impact Questionnaire (FIQ) [32]. The FIQ contains 10 subscales of disabilities and symptoms, ranging from 0 to 10. A total score may be obtained after normalization of some subscales and summing the subscales, the FIQ total score, range from 0 to 100, in which a higher score indicates a greater impact of the syndrome. The FIQ total score, and the subscales for physical function, feel good, pain, fatigue, morning tiredness, stiffness, anxiety, and depression were applied in the study.

Quality of life was assessed by means of the Spanish version of the Short-Form Health Survey 36 (SF-36) [33]. The SF-36 contains 36 statements grouped into 8 subscales: physical functioning, physical role, bodily pain, general health, vitality, social functioning, emotional role, and mental health. The range of scores goes between 0 and 100 in every subscale, in which higher scores indicate better health.

The Spanish version of the Hospital Anxiety and Depression Scale (HADS) [34] was used to assess anxiety and depression. The HADS contains 14 statements, ranging from 0 to 3, in which a higher score indicates a higher degree of distress. The scores build 2 subscales: anxiety (0–21) and depression (0–21) [35].

The Spanish version of the Vanderbilt Pain Management Inventory (VPMI) [36] was used to assess coping strategies. The VPMI contains 18 statements divided into two subscales designed to assess how often chronic pain sufferers use active and passive coping strategies [37].

The Rosenberg Self-Esteem Scale (RSES) is a self-report measure designed to assess the concept of global self-esteem [36, 37]. The RSES comprises just 10 items scored on a 4-point scale that are summed to produce a single index of self-esteem. In this study we used the Spanish version [38, 39].

The Spanish version of the General Self-Efficacy Scale [40] was used to assess the individual beliefs in her/his own capabilities to attain aims. This instrument contains 10 items scored on a 4-point Likert scale from 1 (not at all true) to 4 (exactly true). In this case, higher scores indicate a higher level of perceived general self-efficacy.

### 2.4. Statistical Analysis

Demographic variables were analysed using descriptive analysis. Because of the small sample size of the data and the nonnormality in the distributions of some variables, traditional multilevel modeling techniques that rely on large sample theory for accurate *P* values were not appropriate. The Friedman Test, a nonparametric technique, was used to assess the training effects on the outcome variables across multiple observations. When Friedman test was significant, differences between two testing time points (pretest versus posttest, pretest versus detraining, posttest versus detraining) were tested with Wilcoxon test.

We performed a perprotocol analysis to study the participants who complied with the study protocol, which was defined as attendance at least 60% of the sessions. Analyses were performed using the Statistical Package for Social Sciences (SPSS, v. 16.0 for WINDOWS; SPSS Inc, Chicago). The differences were considered significant for *P* < 0.05.

## 3. Result

Five women discontinued the program due to health problem, and personal conflict, and four women were not included in the final analysis because they did not assist to any of the assessment sessions. Five women were not included in the final analysis for attending less than 60% of the program (attendance 15.56%). Adherence to the intervention was 79.8% (range 61–94%). A total of 23 women with FM completed the 28-week followup. There were no mayor adverse effects and no major health problems in the patients during the training and detraining periods.

Sociodemographic characteristics of women with FM are shown in Table 1.

The effects of Tai-Chi training on pain are showed in the Table 2. We observed significant changes on pain threshold of all the tender points (*P* < 0.001), tender point count (*P* < 0.001), and algometer score (*P* < 0.001). Post hoc analysis revealed that the pain threshold of all the tender points, tender point count, and algometer score significantly improved from pretest to posttest. These changes were maintained after detraining phase (posttest-retest) (Table 2).

Significant changes for sit and reach (*P* < 0.001), back scratch (*P* = 0.002), handgrip strength (*P* = 0.006), chair stand (*P* < 0.001), 8 feet up and go (*P* < 0.001), blind flamingo (*P* < 0.001), and 6-min walk (*P* = 0.006) tests were identified. Post hoc analysis revealed that these functional capacity tests improved from pretest to posttest. The positive changes were not maintained after detraining phase in functional capacity although the scores on handgrip strength, chair stand, 8-feet up and go, blind flamingo and 6-min walk tests were better than the pretest score. Indeed these tests showed significant improvements from pretest to retest (Table 3).

In addition, we found significant changes in FIQ total score (*P* < 0.001) and in six FIQ-subscales: pain (*P* < 0.001), fatigue (*P* < 0.001), morning tiredness (*P* < 0.001), stiffness (*P* = 0.005), anxiety (*P* < 0.001), and depression (*P* < 0.001). Post hoc analysis revealed that the FIQ total score and the FIQ-subscales decreased (positive) from pretest to posttest. These positive changes in FIQ total scores and in the six FIQ-subscales were not maintained after detraining phase, but the FIQ-subscales fatigue, morning tiredness, anxiety, and depression decreased from pretest to retest (Table 4). The statistical analysis showed changes in the following SF-36-subscales: physical function (*P* < 0.001), physical role (*P* < 0.001), bodily pain (*P* = 0.003), general health (*P* < 0.001), vitality (*P* = 0.018) and mental health (*P* < 0.001). Post hoc analysis revealed that the SF-36-subscale physical function, physical role, bodily pain, general health, vitality, and mental health increased (positive) from pretest to posttest. These improvements were maintained after detraining phase in all the previous SF-36-subscales except physical role (Table 4).

We observed changes in the VPMI-active coping subscale (*P* = 0.019) as well as in HADS-depression (*P* < 0.001) and HADS-anxiety (*P* = 0.009) subscales. Post hoc analysis revealed that these variables improved from pretest to posttest, but only the improvement in VPMI-active coping subscale was maintained after detraining phase (Table 5).

We found changes on Self-Efficacy Scale and RSES scores (*P* < 0.001 and *P* < 0.005 resp.). Post hoc analysis revealed that both variables improved from pretest to posttest. These improvements were not maintained after detraining phase, but the detraining scores were better than pretest score, and significant improvements from pretest to retest were identified (Table 4).

## 4. Discussion

This study shows that Tai-Chi exercise is potentially a useful therapy for women with FM. The main finding of the present study is that 28-week Tai-Chi training improved pain and functional capacity. The effects of Tai-Chi training were evident on symptomatology, depression, quality of life, active coping, self-esteem, and self-efficacy. The improvements persisted after the detraining phase in pain threshold, tender points count, algometer score, SF-36 subscales (physical functioning, bodily pain, general health, vitality, emotional role and mental health) and in VPMI-active coping subscale. The program was well tolerated and had not any deleterious effects on the patients' health.

The improvement in lower body flexibility concurs with our previous study [22] performed in 6 men with FM (52.3 ± 9.3 years). In that study, we found a positive change in lower body flexibility after 16-week Tai-Chi training (3 times/week) that was maintained after 12 weeks of detraining period. However, in the present study, the gains of flexibility were not maintained after detraining period.

The results of this study suggest that a Tai-Chi long-term training could be an effective therapy for FIQ total score and the FIQ subscales: pain, fatigue, morning tiredness, stiffness, anxiety, and depression, as well as, on the following SF-36 subscales: physical function, physical role, bodily pain, general health, vitality, and mental health. Similarly, in a recent study, Wang et al. [24] observed improvements on the FIQ total score, and on mental and physical component of the SF-36 after 12 weeks of Tai-Chi training (2 times/week) in 33 women with FM (49.7 ± 11.8 years). On the other hand, this study did not report the SF-36 and FIQ subscales scores or tender point count, and the assessment of functional test is very limited.

Similarly, Taggart et al. [23] found significant changes on the FIQ subscale physical functioning, feel good, pain, morning tiredness, stiffness, and anxiety and in the SF-36 subscales physical functioning, bodily pain, general health, vitality, and emotional role, after 6 weeks of Tai-Chi training (2 times/week) in 21 FM patients (56.2 ± 11.9 years). However, this study did not show the effects of the Tai-Chi training in functional test or tender-point count.

In our study, the FM patients were able to walk greater distances (~40 meters) after Tai-Chi training on 6-min walk test. This finding concurs with the study of Wang et al. [24] that also observed improvements on this test (~55.4 meters).

To note is that this is the first study that analyzed the effect of long-term Tai-Chi training in female FM patients and it is difficult to compare our results with those of other previous studies. However, the results of present study concur with other studies that have analyzed the effects of long-term Tai-Chi training in other diseases.

We observed improvements on aerobic capacity, dynamic balance, and lower-body strength. Similarly, Lan et al. [41] found improvement in cardiorespiratory function in 9 adults patients with coronary artery bypass (56.5 ± 7.4 years) after 52 weeks of Tai-Chi training (45 minutes/every days). Lan et al. [42] found improvements in aerobic capacity after 52 weeks of Tai-Chi training (3 times/week) in 53 patients (52.8 ± 9.4 years) with dyslipidemia. Likewise, Li and Manor [43] observed improvements on aerobic capacity, dynamic balance, and lower-body strength in 25 patients (71±12 years) with peripheral neuropathy after 24 weeks (3 times/week) Tai-Chi training. However, it should be noted that these studies did not perform a detraining phase.

The biologic mechanics by which Tai-Chi might affect the clinical course of FM remains to be known. However, the degree of flexion at the hips and knees [44] the constantly shift weight from one foot to the other, as well as the rotational movements of the head, trunk, and extremities [45, 46] performed during Tai-Chi practice could be related with the improvements found in strength, balance, and flexibility in our study. The interesting findings of the present study should be interpreted in the context of the following limitations. We were not able to conduct a randomized controlled trial with a control group, and it was not possible to control the changes in the FM pharmacological treatment during the training. In addition, we did not control the influence of preexisting beliefs and expectations with respect to Tai-Chi in FM patients. On the other hand, we have analyzed the effects of Tai-Chi training in variables that have not been previously explored in female FM patients, such as tender points, balance, flexibility, strength, depression, coping strategies, self-esteem, and self-efficacy. Moreover, our study analyzed the effects of 12 weeks detraining phase.

The effects of long-term Tai-Chi training need further randomized controlled trials especially focused in the biologic and psychological mechanics by which Tai-Chi exercise might affect the clinical course of FM.

## 5. Conclusions

The improvements observed in pain, symptomatology, functional capacity, quality of life, psychological outcomes, pain-coping strategies, self-esteem and self-efficacy in women with FM after a 28-week Tai-Chi training indicate that Tai-Chi may be a useful and feasible treatment in the management of FM.

## Figures and Tables

**Table 1:** Sociodemographic characteristics of women with FM.

	Tai-Chi group (*n* = 23)
Age, years	51.35 (6.753)
Years since clinical diagnosis, *n* (%)^a^	
≤5 years	11 (52.4)
>5 years	10 (47.6)
Marital status, *n* (%)^b^	
Married	19 (86.4)
Unmarried	1 (4.5)
Separated/divorced/widowed	2 (9.1)
Educational status, *n* (%)^c^	
Unfinished studies	2 (9.1)
Primary school	14 (63.6)
Secondary school	4 (18.2)
University degree	2 (9.1)
Occupational status, *n* (%)^d^	
Housewife	13 (65.0)
Student	1 (5.0)
Working	2 (10.0)
Unemployed	3 (15.0)
Retired	1 (5.0)
Income, *n* (%)^e^	
<1200,00€	13 (76.5)
1200,00–1800.00€	3 (17.6)
>1800,00€	1 (5.9)
Menstruation, *n* (%)^f^	
Yes	7 (33.3)
No	14 (66.7)

a: two missing. b: one missing. c: one missing. d: three missing. e: six missing. f: two missing.

**Table 2:** Effects of 28-week Tai-Chi intervention on tender points.

	Pretest	Posttest	Detraining	*P*	Prepost	Preretest	Postretest
Occiput R	1.49 (0.51)	2.43 (0.65)	2.56 (0.58)	<0.001	<0.001	<0.001	0.172
Occiput L	1.57 (0.49)	2.43 (0.61)	2.49 (0.72)	<0.001	<0.001	<0.001	0.526
Anterior cervical R	1.17 (0.28)	1.72 (0.40)	1.66 (0.47)	<0.001	<0.001	<0.001	0.474
Anterior cervical L	1.17 (0.24)	1.78 (0.43)	1.81 (0.47)	<0.001	<0.001	<0.001	0.767
Trapezius R	1.81 (0.64)	2.78 (0.72)	2.94 (0.67)	<0.001	<0.001	<0.001	0.218
Trapezius L	1.68 (0.64)	2.87 (0.64)	3.03 (0.59)	<0.001	<0.001	<0.001	0.287
Supraspinatus R	1.77 (0.88)	3.19 (1.01)	3.10 (0.83)	<0.001	<0.001	<0.001	0.761
Supraspinatus L	1.90 (0.89)	3.36 (1.07)	3.09 (0.86)	<0.001	<0.001	<0.001	0.196
Second rib R	1.42 (0.40)	2.45 (0.74)	2.44 (0.70)	<0.001	<0.001	<0.001	0.808
Second rib L	1.39 (0.41)	2.68 (1.01)	2.91 (2.56)	<0.001	<0.001	<0.001	0.273
Lateral epicondyle R	1.72 (0.59)	3.37 (0.88)	3.27 (1.21)	<0.001	<0.001	<0.001	0.369
Lateral epicondyle L	1.74(0.59)	3.50 (1.28)	3.75 (1.51)	<0.001	<0.001	<0.001	0.420
Gluteal R	1.76 (0.87)	3.38 (0.88)	3.06 (0.93)	<0.001	<0.001	<0.001	0.088
Gluteal L	1.85 (0.78)	3.33 (1.00)	3.02 (1.01)	<0.001	<0.001	<0.001	0.100
Great trochanter R	1.84 (0.72)	3.68 (1.01)	3.55 (1.31)	<0.001	<0.001	<0.001	0.361
Great trochanter L	1.79 (0.78)	3.62 (1.08)	3.40 (1.14)	<0.001	<0.001	<0.001	0.236
Knee R	1.52 (0.50)	2.93 (0.75)	2.93 (0.94)	<0.001	<0.001	<0.001	0.976
Knee L	1.58 (0.54)	2.99 (0.66)	2.78 (0.78)	<0.001	<0.001	<0.001	0.071
Algometer score	28.86 (8.81)	52.81 (12.10)	52.00 (13.84)	<0.001	<0.001	0.010	0.581

Total number of points	17.91 (0.43)	15.50 (3.21)	16.36 (2.70)	<0.001	0.003	<0.001	0.071

Note. Data are presented as means (standard deviation). R: right; L: left.

**Table 3:** Effects of 28-week Tai-Chi intervention on physical function.

	Pretest	Posttest	Detraining	*P*	Prepost	Preretest	Postretest
Weight (kg)	68.44 (12.27)	69.12 (12.60)	69.00 (12.67)	0.790	0.438	0.721	0.728
Waist circumference (cm)	90.85 (18.39)	87.64 (11.89)	84.82 (12.92)	0.100	0.404	0.008	0.015
BMI (kg/m^2^)	27.41 (4.78)	27.67 (4.70)	28.08 (5.29)	0.781	0.429	0.404	0.970
Chair sit and reach (cm)	−11.84(13.53)	0.80 (12.62)	−5.74 (15.09)	<0.001	<0.001	0.064	<0.001
Back scratch test (cm)	−10.34 (13.65)	−6.25 (10.11)	−10.17 (13.15)	0.002	0.009	0.702	0.002
Handgrip strength (kg)	16.31 (6.50)	20.83 (6.58)	18.07 (6.60)	0.006	<0.001	0.048	0.003
Chair stand test (*n*)	6.68 (1.89)	13.11 (2.71)	10.58 (2.43)	<0.001	<0.001	<0.001	<0.001
8-feet up and go (*s*)	10.45 (2.04)	6.43 (2.03)	6.86 (1.75)	<0.001	<0.001	<0.001	0.008
Blind flamingo (failures)	9.58 (5.01)	5.23 (4.25)	6.65 (4.42)	<0.001	<0.001	<0.001	0.008
6-min walk (*m*)	442.67 (94.71)	481.10 (74.05)	457.42 (62.72)	0.006	0.023	<0.001	0.002

Note. Data are presented as means (standard deviation). BMI: body mass index.

**Table 4:** Effects of 28-week Tai-Chi intervention on symptomatology.

	Pretest	Posttest	Detraining	*P*	Prepost	Preretest	Postretest
FIQ							
Total score	68.58 (8.94)	56.90 (14.52)	67.10 (17.83)	<0.001	<0.001	0.951	0.002
Physical function	4.53 (1.78)	3.63 (1.74)	3.81 (2.35)	0.335	0.101	0.170	0.573
Feel good	6.48 (2.86)	5.98 (2.78)	6.97 (2.52)	0.101	0.397	0.959	0.106
VAS pain	7.64 (1.64)	5.79 (2.20)	7.04 (2.26)	<0.001	<0.001	0.064	<0.001
VAS fatigue	9.00 (0.79)	6.51 (1.70)	7.59 (2.14)	<0.001	<0.001	0.003	0.008
VAS morning tiredness	9.15 (0.87)	6.87 (1.94)	7.96 (2.08)	<0.001	<0.001	<0.001	0.009
VAS stiffness	8.17 (1.60)	6.43 (2.22)	7.54 (2.16)	0.005	0.003	0.083	0.013
VAS anxiety	7.63 (2.32)	5.10 (2.77)	6.29 (2.41)	<0.001	<0.001	0.010	0.013
VAS depression	6.97 (2.63)	4.85 (2.44)	5.91 (2.72)	<0.001	<0.001	0.020	0.035

Note. Data are presented as means (standard deviation). FIQ: Fibromyalgia Impact Questionnaire; VAS: Visual Analogue Scale.

**Table 5:** Effects of 28-week Tai-Chi intervention on quality of life, pain-coping strategies, anxiety, depression, self-efficacy, and self-esteem.

	Pretest	Posttest	Detraining	*P*	Prepost	Preretest	Postretest
*SF-36*							
Physical function	30.87 (13.54)	48.04 (18.93)	46.96 (22.04)	<0.001	<0.001	<0.001	0.519
Physical role	0.00 (0.00)	25.00 (31.81)	7.96 (22.34)	<0.001	0.004	0.059	0.009
Bodily pain	18.86 (15.37)	38.07 (19.12)	28.86 (22.44)	0.003	0.005	0.034	0.080
General health	23.91 (11.87)	36.96 (16.43)	33.26 (18.38)	<0.001	<0.001	0.010	0.190
Vitality	21.30 (12.81)	36.74 (16.90)	29.13 (23.24)	0.018	<0.001	0.148	0.087
Social functioning	45.22 (26.95)	56.74 (20.69)	46.52 (25.57)	0.044	0.085	0.602	0.033
Emotional role	28.79 (41.53)	48.49 (47.95)	33.33 (42.42)	0.138	0.100	0.684	0.125
Mental health	43.83 (22.11)	61.57 (20.88)	56.35 (24.71)	<0.001	0.002	0.012	0.115
*VPMI*							
Passive coping	25.30 (4.42)	22.17 (4.42)	21.96 (6.00)	0.068	0.015	0.017	0.714
Active coping	15.52 (4.31)	18.00 (3.94)	16.70 (4.55)	0.019	0.013	0.078	0.169
*HADS*							
Anxiety	11.43 (4.20)	8.39 (4.34)	10.35 (5.18)	0.009	0.004	0.250	0.020
Depression	9.57 (4.37)	5.91 (3.13)	8.48 (4.60)	<0.001	<0.001	0.055	0.009
*SELF-EFFICACY*	24.70 (6.52)	30.78 (5.59)	28.57 (5.84)	<0.001	<0.001	<0.001	0.043
*RSES*	28.00 (4.90)	31.74 (3.96)	29.30 (5.95)	0.005	0.002	0.056	0.013

Note. Data are presented as means (standard deviation). SF-36: Short Form Health Survey 36; VPMI: Vanderbilt Pain Management Inventory; HADS: Hospital Anxiety and Depression Scale; SELF-EFFICACY: General Self -Efficacy Scale; RSES: Rosenberg Self-Esteem Scale.
